# Effectiveness and impact of networked communication interventions in young people with mental health conditions: A rapid review

**DOI:** 10.1177/2055207618762209

**Published:** 2018-03-21

**Authors:** Alice Verran, Ayesha Uddin, Rachel Court, Frances Taggart, Paul Sutcliffe, Jackie Sturt, Frances Griffiths, Helen Atherton

**Affiliations:** 1 12212 Warwick Medical School, University of Warwick, UK; 2 The Florence Nightingale Faculty of Nursing & Midwifery, King's College London, UK; 3 University of the Witwatersrand, South Africa

**Keywords:** Young adult, transition to adult care, mental health, remote consultation, communication

## Abstract

**Objective:**

To describe the latest evidence of effectiveness and impact of networked communication interventions for young people with mental health conditions.

**Methods:**

Searching five databases from 2009 onwards, we included studies of any design investigating two-way communication interventions for the treatment of young people (mean age 12–25) with a chronic mental health disorder. The data were synthesised using narrative summary.

**Results:**

Six studies met the inclusion criteria, covering a range of mental health conditions (depression, psychosis, OCD). Interventions included an online chat room (*n* = 2), videoconferencing (*n* = 3) and telephone (*n* = 1). Where studies compared two groups, equivalence or a statistically significant improvement in symptoms was observed compared to control. Views of patients and clinicians included impact on the patient-clinician interaction. Clinicians did not feel it hindered their diagnostic ability.

**Conclusion:**

Networked communication technologies show promise in the treatment of young people with mental health problems but the current available evidence remains limited and the evidence base has not advanced much since the previous inception of this review in 2011.

**Practice implications:**

Although the available research is generally positive, robust evidence relating to the provision of care for young persons via these technologies is lacking and healthcare providers should be mindful of this.

## Introduction

Worldwide, mental health conditions are a significant source of disease burden.^1^ The costs are high both on a macro level, with high economic costs,^2^ and on an individual level correlating with poor physical health and emotional wellbeing, lower educational attainment and decreased social capital.^3–5^ In young people, this is particularly significant over the life-course. Many mental health problems begin in childhood or young adulthood,^6^ and their prevalence increases with age, affecting 7.7% of 5–10 year olds, 11.5% of 11–16 year olds and around 23% of 18–20 year olds.^7,8^ Mental health conditions may become chronic and there is evidence to show that early intervention and good care are cost effective and can improve both short and long-term outcomes.^9^

There are a number of challenges associated with providing health care for young people. They face multiple barriers to access including insufficient training of healthcare providers and issues around confidentiality when navigating young adulthood.^10,11^ This is exacerbated by behavioural issues such as risk-taking behaviour and problems transitioning from paediatric to adult services.^12^ There is scope to consider how these barriers can be overcome to facilitate service provision and reduce the development of long-term problems.

The use of networked communication technologies is increasingly widespread, particularly amongst young people and adolescents. In the UK, 91% of 16–24-year-olds own a smartphone,^13^ giving them access to text messaging, email, social networking and videoconferencing in any location where there is network coverage. Health professionals working with young people with mental health conditions are increasingly using digital communication to engage with them, using both one way and two-way communication channels.^14^ This review examines the impact of the use of two-way communication between health professionals and young people with mental health conditions.

To date, only one review has described the effectiveness and impact of networked communication interventions specifically in young people with mental health conditions.^15^ This review found just 12 studies which investigated the use of email and/or web-based electronic diaries, videoconferencing and email communication. Email consultation was linked with symptom improvement and patients showed willingness to use networked communications, but overall the results were inconclusive due to the small number of studies and varied outcomes. As the field of digital communication is swiftly evolving, the current review will build on this previous work, including only literature published since the search date from this previous review (2009), as part of a series of rapid reviews for the LYNC study. The LYNC study explored the use of two-way digital communication technologies in healthcare provision for young people (aged 16–24 years) with long term conditions,^14^ and the rapid reviews were conducted as a means to support and add value to the case studies within the LYNC study and to place the case study findings in a wider research context.

## Methods

The review aims to present evidence for the effectiveness and impact of digital networked communication interventions in the treatment of adolescents with mental health disorders. A rapid review differs from a traditional systematic review primarily in the time taken to complete the review; a rapid review is often conducted within eight weeks.^16^ Rapid reviews must, however, adhere to the core principles of systematic review. The protocol of the rapid review was registered in the International PROSPERO database under the following number: CRD42016038792.

### Population

Our population was adolescents or young adults (mean age 12–25 years) with a mental health condition as defined by the Diagnostic and Statistical Manual of Mental Disorders (DSM-IVTR).^17^ We did not include participants with neurodevelopmental disorders or non-chronic mental health conditions.

### Intervention

The intervention was networked communication technology (e.g. telephone, email, text message and videoconferencing) allowing two-way communication between the patient and healthcare professional. Computerised cognitive behavioural therapy (CCBT) interventions were not included where the focus of the study was the automated computer program. We did not include interventions that allowed only one-way communication.

### Comparator

We included comparison with usual care.

### Outcomes

We included clinical outcomes, patient outcomes and clinician outcomes.

### Inclusion criteria

This review looked at publications from May 2009 onwards. A previous review, published in 2011, searched from inception of databases to 2009.^15^ This review updates the literature since that period. We included any study design and studies in any language. Conference abstracts were excluded unless the abstract itself contained sufficient data to be included in the analysis.

### Search strategy

The search strategy was developed by an information specialist (RC). As part of a series of rapid reviews within the wider LYNC project, a common search was conducted within five electronic databases (MEDLINE (Ovid), Medline In-process (Ovid), Embase (Ovid), PsycINFO (Proquest), Cochrane Library (Wiley)) using free-text and thesaurus terms for the concepts ‘*technology*’, ‘*clinical communication*’ and ‘*population*’ (see Appendix for search strategy). The searches were undertaken in August and September 2015. Records were exported to a bibliographic management software (EndNote X7) and a relevant subset identified by searching within this list for specific conditions and terms relating to ‘mental health’.

### Screening

All titles and abstracts resulting from this search were screened for inclusion by one of two reviewers (AV or AU). A random selection of 20% was screened by both. A third reviewer (FT) resolved any disagreements. Reasons for exclusion were recorded.

Articles that met these criteria after the initial screen were screened as full texts by two reviewers (AV and AU). Any discrepancies over inclusion were resolved through discussion or with the help of a third reviewer (HA).

### Data extraction and quality assessment

Data were extracted from the included studies using a data collection form which collected information on population, intervention, setting, comparator and outcomes. All included papers were assessed for quality using the appropriate Critical Appraisal Skills Programme (CASP) critical appraisal instrument for the study design.^18^ Studies with no limitations identified during this process were classed as ‘good’. Studies with one or two limitations were classed as ‘fair’, and any research with three or more flaws as ‘weak’.

### Data synthesis

Due to the variation in study design and technology type included we present the findings using a narrative summary.

## Results

**Figure 1.**
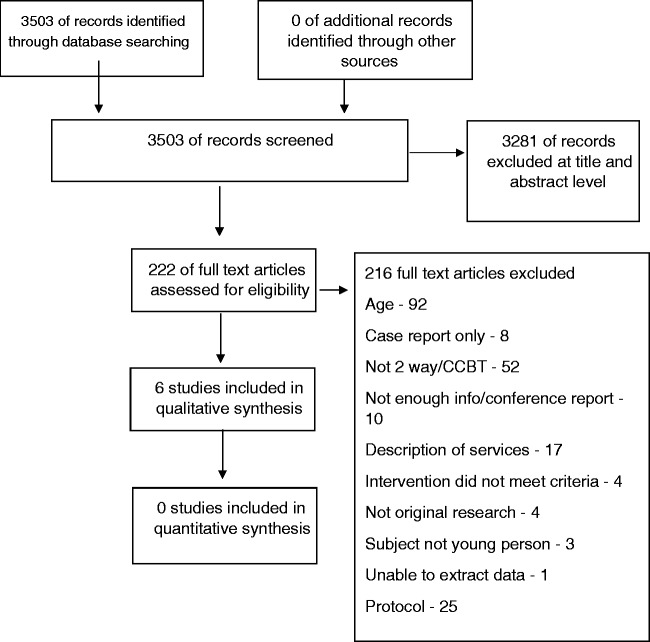
Study flow diagram.

The search identified 3503 articles. 3281 were excluded based on title or abstract. 222 articles were read in full and a total of six studies met our inclusion criteria and formed part of the review.^19–24^ See Figure 1 for a flow chart.

The six studies covered a range of mental health conditions (depression = 3, psychosis = 1, obsessive compulsive disorder (OCD) = 1 and mixed = 1). Interventions included cognitive behavioural therapy (CBT), solution-focused brief therapy (SFBT) and psychiatric consultations. One study looked at neuropsychiatric testing. Two of the interventions used online chat rooms, three used videoconferencing and one used telephone. One intervention was group-based (online chat room) and all others were one-to-one. Studies were located in the USA, UK, Netherlands and Canada. All were published in English. Three randomised trials, one qualitative study and two feasibility studies were included. See Table 1 for summary of study details.

**Table 1.** Characteristics of included studies.

Authors	Country	Study design	Population	Technology and comparator (where relevant)	Outcomes
Boydell et al.^19^	Canada	Qualitative interview study.	Patients receiving psychiatric care via televideo Mean age not given, age range 7–18	Videoconferencing	Patient experience of receiving services
Kramer et al.^20^	The Netherlands	Randomised controlled trial	Young adults and adolescents with depressive symptoms. Mean age 19.5	Web based chatroom versus wait list.	CES-D Score
Stain et al.^21^	USA	Pilot study of feasibility	Patients with psychosis requiring neuropsychological testing Mean age 20 years	Videoconferencing	Neuropsychological tests: WTAR, WMS-R Logical Memory Subtest; WAIS-III Digit Span Subtest, COWA Clinical tests: BPRS, AQL, SOFAS
Turner et al.^22^	UK	Randomised controlled non-inferiority trial	Adolescents with OCD Mean age 14 years	Telephone cognitive behavioural therapy versus face-to-face	Primary: CY-BOCS Secondary: depressive symptoms, self-report, and parent-report of adolescent OCD symptoms, overall psychological health, global functioning, and parental mental health symptoms
Van der Zanden et al.^23^	The Netherlands	Randomised trial	Young people with depressive symptoms Mean not given, range 16–25	Online chatroom as part of online group-based CBT versus waitlist	Primary: CES-D score. Secondary: HADS, 5-item mastery scale (sense of control)
Williams et al.^24^	USA	Feasibility and acceptability study using survey assessment.	Students with depression at universities in US Mean age not given, participants are ‘college students’	Videoconferencing	Satisfaction and technological issues.

Overall the quality of included papers was fair. Only one paper was classed as good;^21^ four were fair,^19,20,22,23^ but one was weak according to our pre-specified criteria.^24^

### Clinical outcomes

Clinical outcomes were reported by four papers (see Table 2).^19–22^ The standardised scores used were the Center for Epidemiologic Studies Depression (CES-D) Scale and the Hospital Anxiety and Depression Scale (HADS). Van der Zanden et al. investigated the effectiveness of group CBT delivered via an online chatroom over six weekly sessions.^23^ They found a statistically significant improvement in depressive symptoms, anxiety and mastery at both 12 weeks (depressive symptoms (CES-D *d* = 0.94, 95% confidence interval (CI) 0.64–1.23), anxiety (HADS *d* = 0.49, 95% CI 0.24–0.75), mastery (*d* = 0.44, 95% CI 0.19–0.70)) and 24 weeks (depressive symptoms (CES-D *d* = 1.13, 95% CI 0.78–1.47), anxiety (HADS d = 0.53, 95% CI 0.25–0.81), mastery (*d* = 0.51, 95% CI 0.23–0.79)) compared to the control group. Kramer et al. also used an online chatroom to deliver their intervention, investigating one-to-one SFBT for depression.^20^ They found an improvement in CES-D scores in the intervention group compared to the waitlist control at 9 weeks (*d* = 0.18, 95% CI −0.10 to 0.47) and 4.5 months (*d* = 0.79, 95% CI 0.45–1.08) but note the 9 week result is not significant. Furthermore, those in the intervention group who did not attend any chat sessions also improved compared to controls (*d* = 1.79 vs *d* = 0.93, *p* < .001).^20^

**Table 2.** Summary of main results.

Authors	Summary of main results
Boydell et al.^19^	30 participants took part in the study. **Patient satisfaction** Participants reported increased knowledge around mental health and coping mechanisms. Benefits included anonymity, the medium being less intimidating and anxiety provoking, and being a novelty. Negatives experiences included experiencing a lack of control and feeling emotionally ‘distanced’ from the encounter.
Kramer et al.^20^	263 patients took part in the study. 131 in the intervention group and 132 in the control group. **CES-D Score** Baseline (mean): Chat – 39.49, Waitlist (WL) – 39.74 9 weeks (mean): Chat – 29.20, WL – 32.51. Between group effect size (*d* = 0.18, 95% CI −0.10 to 0.47) 4.5 months (mean): Chat – 24.86, WL – 33.09. Between group effect size of *d* = 0.79 (95% CI 0.45–1.08). 7.5 months (mean): Chat – 20.31
Stain et al.^21^	11 participants took part in the study **Neuropsychological and clinical tests using a range of measures.** Most tests demonstrated equivalence (SOFAS, WTAR, COWAT, BPRS, Logical Memory subtest of the Wechsler Memory Scale) Difficulties were encountered when administering the AQoL by videoconference due to the multiple-choice format.
Turner et al.^22^	72 participants took part in the study. 36 in the intervention group and 36 in the control group. **Primary outcomes: CY-BOCS** Intent-to-treat analyses showed that TCBT was not inferior to face-to-face CBT at posttreatment, 3-month, and 6-month follow-up. At 12-month follow-up, there were no significant between-group differences for the CY-BOCS, however the confidence intervals exceeded the non-inferiority threshold. Improvements made during treatment were maintained through to 12-month follow-up **Secondary outcomes: depressive symptoms, self-report and parent-report of adolescent OCD symptoms, overall psychological health, global functioning, and parental mental health symptoms.** All secondary measures confirmed non-inferiority at all assessment points.

The delivery of CBT via telephone was investigated by Turner et al.^22^ They compared telephone CBT to face-to-face CBT for adolescents with OCD and found no significant clinical difference between the two groups following treatment after assessment with several clinical scales (Children's Yale–Brown Obsessive-Compulsive Scale (CY-BOCS), Children's Obsessional Compulsive Inventory–Revised (ChOCI-R), Beck Depression Inventory for Youth (BDI-Y), Strengths and Difficulties Questionnaire (SDQ)). Their findings indicated non-inferiority of telephone CBT compared to face-to-face treatment.^22^

Stain et al. investigated the feasibility of neuropsychological testing via videoconferencing in adolescents with early psychosis.^21^ Most tests demonstrated equivalence (Social and Occupational Functioning Assessment Scale (SOFAS), Wechsler Test of Adult Reading (WTAR), the Controlled Oral Word Association Test (COWAT), the Logical Memory subtest of the Wechsler Memory Scale and the BPRS) indicating that they can reliably be carried out by videoconferencing. Difficulties were encountered when administering the Assessment of Quality of Life (AQoL), however, owing to the multiple-choice format.^21^

Two qualitative interview studies gave an insight into the patient experience of psychiatric consultations conducted by videoconferencing (see Table 2).^19,24^ It is important to note that both these papers reported on patient experience after a single telepsychiatric consultation only. Participants generally found the consultations useful and they were reported to increase knowledge of the mental health disorder and coping methods. Some appreciated the consultation taking place at home where they felt comfortable, and also the anonymity provided by the clinician's distance. This separation made the encounter less stressful and intimidating for some, while for others it stimulated less engagement. Members of the younger group reported excitement at the novelty but also a lack of control. They were required to have an adult in the room with them during the consultation. Issues with sound or video quality were reported but these were generally brief and seemed not to interfere with the overall consultation.

One study reported that clinicians felt confident in making their diagnosis via videoconferencing.^24^

## Discussion and conclusion

### Discussion

This rapid review found that networked communication technologies for the treatment of mental health disorders are generally acceptable to young people. It included data on a range of technologies including videoconferencing, telephone and online chatrooms. Due to the clinical and methodological heterogeneity between the included studies, it was not possible to combine results relating to individual technologies or a specific mental health disorder, which would be useful in drawing robust conclusions. We found evidence relating to a range of mental health disorders (anxiety, depression, psychosis, OCD).

Two studies demonstrated the equivalence of interventions delivered via telephone and videoconferencing as compared to face-to-face.^21,22^ Another study reported improved clinical outcomes compared to waitlist controls in those who received online group CBT.^23^ Together, this seems to indicate networked communication technologies could be a viable alternative to standard face-to-face therapy.

In the earlier version of this review, which examined studies up until 2009,^15^ networked communication technologies for the treatment of mental health problems were generally positive, but there were only 12 studies identified. This latest version of the review noted fewer reservations about privacy, with participants instead reporting the distance enabled them to feel more anonymous.^19^ This could perhaps reflect an increasing trust in technology over time. Furthermore, equipment quality seemed to have improved since the previous study and significantly fewer issues were reported.

Other previous reviews looking at the use of communication technologies within mental healthcare for young people have generally been broad in their inclusion criteria. Many are dominated by CCBT, perhaps because of its ready uptake by healthcare providers. Example of this are the studies by Ye et al. (anxiety or depression) and Aardoom et al. (eating disorders),^25,26^ in which the majority of included articles focus on CCBT (6/7 articles and 14/21 respectively). Nevertheless, few reviews have looked specifically at CCBT for the treatment of young people. Those that have are generally positive in their findings but indicate that further research is needed.^27,28^ In contrast, this review has looked exclusively at two-way communication and has therefore excluded interventions such as CCBT.

Two-way communication is more similar to current clinical treatments for mental health disorders such as counselling and psychotherapy but assumptions about its equivalence are unwise. Different technologies involve different levels of human contact (compare videoconferencing to a text message) and it seems possible that effectiveness may vary with the method used.

A 2014 rapid review of interventions in ‘e-mental health’ took a broad approach and found that the majority of interventions were aimed at adults with depression or anxiety symptoms. Effectiveness was demonstrated in some early trials and other studies summarised benefits that included flexibility and engagement.^29^ However, this review focused on adult populations and was not restricted to two-way communication. A review and meta-analysis of e-mental health interventions to treat post-traumatic stress disorder (PTSD) in adults showed a significant improvement in symptoms for those using the e-mental health interventions. However, this study included interventions that had no communication, or one-way communication.^30^

Overall, the evidence available regarding young people and their use of two-way networked digital communications remains limited. Nevertheless, findings are generally positive with studies reporting improved symptoms scores or equivalence to current treatment. It is unclear whether drop out might be an issue for this particular young population. Drop out is known to be lower when interventions are delivered online.^31^ However there is no age specific data and so it is not possible to assess whether this might be a particular issue for those in adolescence and young adulthood.

This review was conducted to inform a wider study looking at networked communication technologies for the treatment of young people.^14^ This gave us an overview of its use in practice and allowed us to see mental health within this context but restricted the time taken in conducting the review. Taking a ‘rapid’ approach meant that grey literature was not included and it is therefore possible that some relevant data may have been missed. Nevertheless, the search was broad and covered a wide range of mental health conditions, included non-English language papers, and was developed by an information specialist. This review examines the more recent literature since publication of the previous version of this review, which was important given the rapid pace of change within digital technologies.

### Conclusion

Networked communication technologies show promise in the treatment of young people with mental health disorders but there are many conditions and methods of treatment that have not yet been fully investigated. The use of communication technology is widespread amongst young people and adolescents and is already becoming more prevalent within healthcare. Research into this important age group has not kept up with policy and practice and evaluation of these approaches is much needed.

### Practice implications

Although there is enthusiasm for the use of communication technology in healthcare with young people, robust evidence is lacking and healthcare providers should be mindful of this. Appropriate use should be considered and steps taken to mitigate patients' concerns about privacy to ensure its positive effects are not negated. Practitioners should bear in mind that the effectiveness of most interventions for the majority of mental health conditions in young people not yet been thoroughly investigated and ample opportunities for further research exist. The use of communication technology within mental healthcare could improve access but ongoing research would be required to ensure these people are not disadvantaged by poorly evidenced treatment.

**Table:** 

1	Electronic Mail/	2038
2	(email* or e-mail* or web-mail* or webmail* or internet-mail*).tw.	7666
3	Text Messaging/	887
4	(text messag* or texting or multimedia message*).tw.	1263
5	((mobile phone* or cellular phone* or cell phone*) and (message* or text* or sms or mms)).tw.	819
6	Social Media/	1923
7	(social media or social networking or blog* or facebook or myspace or twitter).tw.	3505
8	((internet* or web* or information or patient or health) adj2 (portal* or forum)).tw.	1673
9	(smartphone app* or smart phone app* or PDA app* or personal digital assistant app*).tw.	331
10	(video-conferenc* or videoconferenc* or videophone* or video-phone* or Voice over Internet Protocol or VoIP or skype or (google adj2 (talk or hangouts))).tw.	1810
11	Videoconferencing/	923
12	digital interactive television.tw.	5
13	1 or 2 or 3 or 4 or 5 or 6 or 7 or 8 or 9 or 10 or 11 or 12	17838
14	(digital or electronic or virtual or computer* or software* or internet* or online or on-line or web* or multimedia or multi-media or communication technolog* or telecommunication* or ICT or network* technolog* or telemedic* or telecare or telehealth* or telepsychiatr*).ti.	163183
15	*computer communication networks/ or *internet/	37023
16	*telecommunications/ or *telemedicine/ or *remote consultation/ or *telepathology/ or *teleradiology/ or *cell phones/ or *modems/ or *wireless technology/	22303
17	13 or 14 or 15 or 16	200778
18	exp *Professional-Patient Relations/ or exp *Professional-Family Relations/	62797
19	((clinic* or center* or centre* or service* or hospital* or doctor* or physician* or clinician* or nurse* or pharmacist* or health worker* or professional* or provider* or practitioner* or therapist* or educator* or psychiatr* or patient* or outpatient* or out-patient* or inpatient* or in-patient* or client* or child* or teen* or paediatric* or pediatric* or boy* or girl* or youth* or schoolchild* or adoles* or minor or minors or under age* or juvenile* or schoolage* or school age* or young adult* or young person* or young people or student* or parent* or mother* or father* or brother* or sister* or sibling* or family or families or carer* or caregiver* or care giver*) adj4 (communicat* or relation* or interact* or convers* or discuss* or message* or feedback or respond* or response* or receive* or consult* or contact* or advice or advis* or counsel* or recommend* or monitor* or review* or diary or diaries or assess* or support* or educat* or train* or manage* or care or treat* or therapy or therapies or intervention* or report* or ongoing partnership)).tw.	2616393
20	18 or 19	2652071
21	17 and 20	41890
22	Young Adult/ or Adolescent/ or Child/ or Students/	2569792
23	(child* or teen* or paediatric* or pediatric* or boy* or girl* or youth* or schoolchild* or school child* or kid* or adoles* or minor or minors or under age* or juvenile* or pubescen* or secondary school* or highschool* or high school* or peer group* or schoolage* or school age* or young adult* or young person* or young people or student* or sixth form* or higher education or further education or undergraduate* or college* or universit*).tw.	2217902
24	22 or 23	3751056
25	21 and 24	15042

**Table:** 

1	Electronic Mail/	0
2	(email* or e-mail* or web-mail* or webmail* or internet-mail*).tw.	1509
3	Text Messaging/	0
4	(text messag* or texting or multimedia message*).tw.	457
5	((mobile phone* or cellular phone* or cell phone*) and (message* or text* or sms or mms)).tw.	257
6	Social Media/	0
7	(social media or social networking or blog* or facebook or myspace or twitter).tw.	1327
8	((internet* or web* or information or patient or health) adj2 (portal* or forum)).tw.	306
9	(smartphone app* or smart phone app* or PDA app* or personal digital assistant app*).tw.	222
10	(video-conferenc* or videoconferenc* or videophone* or video-phone* or Voice over Internet Protocol or VoIP or skype or (google adj2 (talk or hangouts))).tw.	240
11	Videoconferencing/	0
12	digital interactive television.tw.	1
13	2 or 4 or 5 or 7 or 8 or 9 or 10 or 12	3888
14	(digital or electronic or virtual or computer* or software* or internet* or online or on-line or web* or multimedia or multi-media or communication technolog* or telecommunication* or ICT or network* technolog* or telemedic* or telecare or telehealth* or telepsychiatr*).ti.	27501
15	13 or 14	30742
16	((clinic* or center* or centre* or service* or hospital* or doctor* or physician* or clinician* or nurse* or pharmacist* or health worker* or professional* or provider* or practitioner* or therapist* or educator* or psychiatr* or patient* or outpatient* or out-patient* or inpatient* or in-patient* or client* or child* or teen* or paediatric* or pediatric* or boy* or girl* or youth* or schoolchild* or adoles* or minor or minors or under age* or juvenile* or schoolage* or school age* or young adult* or young person* or young people or student* or parent* or mother* or father* or brother* or sister* or sibling* or family or families or carer* or caregiver* or care giver*) adj4 (communicat* or relation* or interact* or convers* or discuss* or message* or feedback or respond* or response* or receive* or consult* or contact* or advice or advis* or counsel* or recommend* or monitor* or review* or diary or diaries or assess* or support* or educat* or train* or manage* or care or treat* or therapy or therapies or intervention* or report* or ongoing partnership)).tw.	252369
17	15 and 16	5700
18	(child* or teen* or paediatric* or pediatric* or boy* or girl* or youth* or schoolchild* or school child* or kid* or adoles* or minor or minors or under age* or juvenile* or pubescen* or secondary school* or highschool* or high school* or peer group* or schoolage* or school age* or young adult* or young person* or young people or student* or sixth form* or higher education or further education or undergraduate* or college* or universit*).tw.	191893
19	17 and 18	1938

**Table:** 

1	*e-mail/	1150
2	(email* or e-mail* or web-mail* or webmail* or internet-mail*).tw.	18033
3	*text messaging/	780
4	(text messag* or texting or multimedia message*).tw.	2242
5	((mobile phone* or cellular phone* or cell phone*) and (message* or text* or sms or mms)).tw.	1469
6	*social media/	2002
7	(social media or social networking or blog* or facebook or myspace or twitter).tw.	6596
8	((internet* or web* or information or patient or health) adj2 (portal* or forum)).tw.	2702
9	(smartphone app* or smart phone app* or PDA app* or personal digital assistant app*).tw.	866
10	(video-conferenc* or videoconferenc* or videophone* or video-phone* or Voice over Internet Protocol or VoIP or skype or (google adj2 (talk or hangouts))).tw.	2742
11	*videoconferencing/	576
12	digital interactive television.tw.	7
13	1 or 2 or 3 or 4 or 5 or 6 or 7 or 8 or 9 or 10 or 11 or 12	33230
14	(digital or electronic or virtual or computer* or software* or internet* or online or on-line or web* or multimedia or multi-media or communication technolog* or telecommunication* or ICT or network* technolog* or telemedic* or telecare or telehealth* or telepsychiatr*).ti.	219504
15	*computer network/ or *internet/	34588
16	exp *telehealth/ or *teleconsultation/ or *mobile phone/	18931
17	13 or 14 or 15 or 16	267290
18	*doctor patient relation/ or *nurse patient relationship/	47263
19	((clinic* or center* or centre* or service* or hospital* or doctor* or physician* or clinician* or nurse* or pharmacist* or health worker* or professional* or provider* or practitioner* or therapist* or educator* or psychiatr* or patient* or outpatient* or out-patient* or inpatient* or in-patient* or client* or child* or teen* or paediatric* or pediatric* or boy* or girl* or youth* or schoolchild* or adoles* or minor or minors or under age* or juvenile* or schoolage* or school age* or young adult* or young person* or young people or student* or parent* or mother* or father* or brother* or sister* or sibling* or family or families or carer* or caregiver* or care giver*) adj4 (communicat* or relation* or interact* or convers* or discuss* or message* or feedback or respond* or response* or receive* or consult* or contact* or advice or advis* or counsel* or recommend* or monitor* or review* or diary or diaries or assess* or support* or educat* or train* or manage* or care or treat* or therapy or therapies or intervention* or report* or ongoing partnership)).tw.	3905370
20	18 or 19	3933505
21	17 and 20	63407
22	exp student/	91612
23	(child* or teen* or paediatric* or pediatric* or boy* or girl* or youth* or schoolchild* or school child* or kid* or adoles* or minor or minors or under age* or juvenile* or pubescen* or secondary school* or highschool* or high school* or peer group* or schoolage* or school age* or young adult* or young person* or young people or student* or sixth form* or higher education or further education or undergraduate* or college* or universit*).tw.	3188530
24	limit 21 to (child < unspecified age > or school child < 7 to 12 years > or adolescent < 13 to 17 years>)	8020
25	22 or 23	3205416
26	21 and 25	20498
27	24 or 26	22140

**Table:** 

#1	MeSH descriptor: [Electronic Mail] this term only	208
#2	(email* or e-mail* or webmail* or web-mail* or internet-mail*):ti,ab,kw	1042
#3	MeSH descriptor: [Text Messaging] this term only	161
#4	(text next messag* or texting or multimedia next message*):ti,ab,kw	529
#5	((mobile next phone* or cellular next phone* or cell next phone*) and (message* or text* or sms or mms)):ti,ab,kw	334
#6	MeSH descriptor: [Social Media] this term only	26
#7	(“social media” or “social networking” or blog* or facebook or myspace or twitter):ti,ab,kw	190
#8	((internet* or web* or information or patient or health) near/2 (portal* or forum)):ti,ab,kw	110
#9	(smartphone next app* or “smart phone” next app* or PDA next app* or “personal digital assistant” next app*):ti,ab,kw	69
#10	(video-conferenc* or videoconferenc* or videophone* or video-phone* or “Voice over Internet Protocol” or VoIP or skype or (google near/2 (talk or hangouts))):ti,ab,kw	318
#11	MeSH descriptor: [Videoconferencing] this term only	98
#12	“digital interactive television”:ti,ab,kw	1
#13	#1 or #2 or #3 or #4 or #5 or #6 or #7 or #8 or #9 or #10 or #11 or #12	2260
#14	(digital or electronic or virtual or computer* or software* or internet* or online or on-line or web* or multimedia or multi-media or communication next technolog* or telecommunication* or ICT or network* next technolog* or telemedic* or telecare or telehealth* or telepsychiatr*):ti	10980
#15	[mh ^“computer communication networks” [mj]] or [mh ^internet [mj]]	1045
#16	[mh ^telecommunications [mj]] or [mh ^telemedicine [mj]] or [mh ^“remote consultation” [mj]] or [mh ^telepathology [mj]] or [mh ^teleradiology [mj]] or [mh ^“cell phones” [mj]] or [mh ^modems [mj]] or [mh ^“wireless technology” [mj]]	528
#17	#13 or #14 or #15 or #16	12873
#18	[mh “Professional-Patient Relations” [mj]] or [mh “Professional-Family Relations” [mj]]	1808
#19	((clinic* or center* or centre* or service* or hospital* or doctor* or physician* or clinician* or nurse* or pharmacist* or health next worker* or professional* or provider* or practitioner* or therapist* or educator* or psychiatr* or patient* or outpatient* or out-patient* or inpatient* or in-patient* or client* or child* or teen* or paediatric* or pediatric* or boy* or girl* or youth* or schoolchild* or adoles* or minor or minors or under next age* or juvenile* or schoolage* or school next age* or young next adult* or young next person* or young next people or student* or parent* or mother* or father* or brother* or sister* or sibling* or family or families or carer* or caregiver* or care next giver*) near/4 (communicat* or relation* or interact* or convers* or discuss* or message* or feedback or respond* or response* or receive* or consult* or contact* or advice or advis* or counsel* or recommend* or monitor* or review* or diary or diaries or assess* or support* or educat* or train* or manage* or care or treat* or therapy or therapies or intervention* or report* or ongoing next partnership)):ti,ab,kw	347291
#20	#18 or #19	347294
#21	#17 and #20	6476
#22	[mh ^“Young Adult”] or [mh ^Adolescent] or [mh ^Child] or [mh ^Students]	78701
#23	(child* or teen* or paediatric* or pediatric* or boy* or girl* or youth* or schoolchild* or school next child* or kid* or adoles* or minor or minors or under next age* or juvenile* or pubescen* or secondary next school* or highschool* or high next school* or peer next group* or schoolage* or school next age* or young next adult* or young next person* or young next people or student* or sixth next form* or higher next education or further next education or undergraduate* or college* or universit*):ti,ab,kw	231913
#24	#22 or #23	231913
#25	#21 and #24	2781

**Table:** 

S24	S17 AND S22 Limited by: Publication date after 2009	2,957*
S23	S17 AND S22	4,707*
S22	S18Limits applied	188,824*
S21	S19 AND S20	9,421*
S20	TI,AB(child* OR teen* OR paediatric* OR pediatric* OR boy* OR girl* OR youth* OR schoolchild* OR school PRE/0 child* OR kid* OR adoles* OR minor OR minors OR under PRE/0 age* OR juvenile* OR pubescen* OR secondary PRE/0 school* OR highschool* OR high PRE/0 school* OR peer PRE/0 group* OR schoolage* OR school PRE/0 age* OR young PRE/0 adult* OR young PRE/0 person* OR young PRE/0 people OR student* OR sixth PRE/0 form* OR higher PRE/0 education OR further PRE/0 education OR undergraduate* OR college* OR universit*)	1,245,671*
S19	S17 AND S18	15,817*
S18	TI,AB((clinic* OR center* OR centre* OR service* OR hospital* OR doctor* OR physician* OR clinician* OR nurse* OR pharmacist* OR health PRE/0 worker* OR professional* OR provider* OR practitioner* OR therapist* OR educator* OR psychiatr* OR patient* OR outpatient* OR out-patient* OR inpatient* OR in-patient* OR client* OR child* OR teen* OR paediatric* OR pediatric* OR boy* OR girl* OR youth* OR schoolchild* OR adoles* OR minor OR minors OR under PRE/0 age* OR juvenile* OR schoolage* OR school PRE/0 age* OR young PRE/0 adult* OR young PRE/0 person* OR young PRE/0 people OR student* OR parent* OR mother* OR father* OR brother* OR sister* OR sibling* OR family OR families OR carer* OR caregiver* OR care PRE/0 giver*) PRE/3 (communicat* OR relation* OR interact* OR convers* OR discuss* OR message* OR feedback OR respond* OR response* OR receive* OR consult* OR contact* OR advice OR advis* OR counsel* OR recommend* OR monitor* OR review* OR diary OR diaries OR assess* OR support* OR educat* OR train* OR manage* OR care OR treat* OR therapy OR therapies OR intervention* OR report* OR ongoing PRE/0 partnership))	642,259*
S17	S10 OR S11 OR S12 OR S13 OR S14 OR S15 OR S16	84,172*
S16	MJSUB.EXACT(“Online Therapy”)	1,492°
S15	MJSUB.EXACT(“Telemedicine”)	2,614°
S14	MJSUB.EXACT(“Internet”)	19,034*
S13	MJSUB.EXACT(“Cellular Phones”)	2,003°
S12	MJSUB.EXACT(“Websites”)	2,602°
S11	TI(digital OR electronic OR virtual OR computer* OR software* OR internet* OR online OR on-line OR web* OR multimedia OR multi-media OR communication PRE/0 technolog* OR telecommunication* OR ICT OR network* PRE/0 technolog* OR telemedic* OR telecare OR telehealth* OR telepsychiatr*)	63,637*
S10	S1 OR S2 OR S3 OR S4 OR S5 OR S6 OR S7 OR S8 OR S9	22,147*
S9	MJSUB.EXACT.EXPLODE(“Electronic Communication”)	10,148*
S8	TI,AB(“digital interactive television”)	4°
S7	TI,AB(video-conferenc* OR videoconferenc* OR videophone* OR video-phone* OR “Voice over Internet Protocol” OR VoIP OR skype OR (google PRE/1 (talk OR hangouts)))	1,466°
S6	TI,AB((smartphone PRE/1 app* OR “smart phone” PRE/1 app* OR PDA PRE/1 app* OR “personal digital assistant” PRE/1 app*))	169°
S5	TI,AB((internet* OR web* OR information OR patient OR health) NEAR/1 (portal* OR forum))	745°
S4	TI,AB(“social media” OR “social networking” OR blog* OR facebook OR myspace OR twitter)	7,850*
S3	TI,AB((mobile PRE/0 phone* OR cellular PRE/0 phone* OR cell PRE/0 phone*) AND (message* OR text* OR sms OR mms))	766°
S2	TI,AB(text PRE/0 messag* OR texting OR multimedia PRE/0 message*)	1,342°
S1	TI,AB(email* OR e-mail* OR web-mail* OR webmail* OR internet-mail*)	6,408*

**Table:** 

Term	Any Field	Title Field
General mental health		
mental*		401
psych*		576
disorder*		418
Specific mental health conditions		
*depress*	1053	
affective	141	
mood*	339	
manic* or mania*	121	
bipolar	64	
cyclothymi*	1	
dysthymi*	4	
schizo*	102	
delusion*	7	
paranoi*	16	
autis*	179	
asperger*	10	
“eating disorder”	122	
“eating disorders”	105	
anorexi*	43	
bulimi*	38	
hyperkin*	3	
hyperactivity	140	
ADHD	128	
attention-deficit	57	
“attention deficit”	167	
self-harm*	36	
“self harm”	11	
“self harms”	0	
“self harming”	0	
“self harmed”	0	
“self harmer”	0	
suicid*	195	
overdos*	30	
PTSD (n.b. Match Case ticked)	50	
post-traumatic	56	
posttraumatic	77	
“post traumatic”	3	
anx*	899	
OCD (n.b. Match Case ticked)	33	
obsessi*	61	
compulsi*	96	
phobi*	134	
panic*	357	
“personality disorder”	44	
“personality disorders”	15	
“behavioural disorder”	0	
“behavioural disorders”	0	
“behavioral disorder”	12	
“behavioral disorders”	10	
“behaviour disorder”	4	
“behaviour disorders”	0	
“behavior disorder”	149	
“behavior disorders”	128	
“conduct disorder”	18	
“conduct disorders”	1	
“body dysmorphia”	0	
“body dysmorphic”	3	

Totals:

N.B. Many records will include more than one of the above terms. Therefore, the totals below are not the sum of the numbers above.

1. Specific condition keywords in any fields: 2835

2. General mental health keywords in title: 1292

3. 1 OR 2 = 3503 (final total for screening)

## Supplemental Material

Appendix -Supplemental material for Effectiveness and impact of networked communication interventions in young people with mental health conditions: A rapid review Click here for additional data file. Supplemental material, Appendix for Effectiveness and impact of networked communication interventions in young people with mental health conditions: A rapid review by Alice Verran, Ayesha Uddin, Rachel Court, Frances Taggart, Paul Sutcliffe, Jackie Sturt, Frances Griffiths and Helen Atherton in Digital Health

## Appendix

### Search strategy

#### Cochrane Library (Wiley), searched 2 September 2015

All results (2781)

Cochrane reviews (39)

Other reviews (61)

Trials (2588)

Methods studies (67)

Technology assessments (6)

Economic evaluations (20)

Cochrane groups (0)

#### PsycINFO (Proquest), searched 8 September 2015

N.B. Results exported from line 24. Line S24 limited to 2009 onwards due to Proquest restrictions on the number of records that can be exported. Unable to export results from line S21.

Results of the above searches were exported to an EndNote Library and de-duplicated. Records were then sorted by year of publication in EndNote and any with 2009 and later or blank in the ‘Year’ field were moved into a group. The following search terms were then applied within this group.
